# F-Actin-Dependent Regulation of NESH Dynamics in Rat Hippocampal Neurons

**DOI:** 10.1371/journal.pone.0034514

**Published:** 2012-04-04

**Authors:** Jeomil Bae, Bong Hwan Sung, In Ha Cho, Woo Keun Song

**Affiliations:** 1 Cell Dynamics and Bioimaging Research Center, School of Life Sciences, Gwangju Institute of Science and Technology, Gwangju, Korea; 2 Department of Cancer Biology, Vanderbilt University Medical Center, Nashville, Tennessee, United States of America; University of Nebraska Medical Center, United States of America

## Abstract

Synaptic plasticity is an important feature of neurons essential for learning and memory. Postsynaptic organization and composition are dynamically remodeled in response to diverse synaptic inputs during synaptic plasticity. During this process, the dynamics and localization of postsynaptic proteins are also precisely regulated. NESH/Abi-3 is a member of the Abl interactor (Abi) protein family. Overexpression of NESH is associated with reduced cell motility and tumor metastasis. Strong evidence of a close relationship between NESH and the actin cytoskeleton has been documented. Although earlier studies have shown that NESH is prominently expressed in the brain, its function and characteristics are yet to be established. Data from the present investigation suggest that synaptic localization of NESH in hippocampal neurons is regulated in an F-actin-dependent manner. The dynamic fraction of NESH in the dendritic spine was analyzed using FRAP (fluorescence recovery after photobleaching). Interestingly, F-actin stabilization and disruption significantly affected the mobile fraction of NESH, possibly through altered interactions of NESH with the F-actin. In addition, NESH was synaptically targeted from the dendritic shaft to spine after induction of chemical LTP (long-term potentiation) and the translocation was dependent on F-actin. Our data collectively support the significance of the F-actin cytoskeleton in synaptic targeting of NESH as well as its dynamics.

## Introduction

Dendritic spines are tiny protrusions that generate most excitatory synapses by receiving synaptic inputs from presynaptic terminals of axons and act as crucial sites of receiving, combining, processing and storing information [1]. Postsynaptic density (PSD) and actin cytoskeleton are the major components of dendritic spines. PSD, an electron-dense structure underlying the postsynaptic membrane, acts as a platform where glutamate receptors, channels, adhesion molecules, scaffolding proteins and signaling proteins cluster at the postsynaptic site [2], [3]. The actin cytoskeleton plays pivotal roles in the formation, maintenance and elimination of dendritic spines, and not only affects the overall structure of spines but also plays key roles in synaptic activity by organizing the postsynaptic density and anchoring postsynaptic receptors to transmit synaptic stimuli [4], [5]. PSD and the actin cytoskeleton in dendritic spines undergo remarkable structure and function remodeling under various synaptic inputs [6]. Remodeling of the dendritic spine is associated with phenomena underlying synaptic strength and plasticity, such as LTP (long-term potentiation) [7], [8]. Information within the brain can be stored by strengthening or weakening synapses, which is mediated by molecular reorganization of postsynaptic components, including PSD constituents and the actin cytoskeleton. These functional and structural changes in dendritic spines and synapse are believed to be the neural basis of learning, memory and cognition in the brain [9], [10].

NESH is the third reported member of the Abi (Abl-interactor) protein family, and hence is also designated Abi-3. NESH was originally identified as a new human gene that possesses a Src homology 3 (SH3) domain, and subsequently included as a member of the Abi family based on its sequence similarity to Abi-1 and -2, which are known regulators of the actin cytoskeleton as well as tumor suppressors [11], [12]. NESH contains several proline-rich motifs in the middle of its sequence and a SH3 domain in the C-terminal region. Ectopic expression of NESH in a metastatic cancer cell line has been shown to suppress cell motility and metastatic potential *in vivo*, possibly mediated through regulation of PAK (p21-activated kinase) [13]. In non-neuronal cells, NESH forms a complex with the actin regulator, WAVE, which is crucial for membrane ruffling and lamellipodia formation [14]. Moreover, NESH interacts with IRSp53 that plays a pivotal role in lamellipodia formation in motile cells by associating with Rac GTPase, and its overexpression blocks PDGF-stimulated membrane ruffling to a significant extent in mammalian cells [15]. These findings collectively suggest that NESH is involved in actin cytoskeleton remodeling during regulation of the cortical membrane and motility. While the molecular and functional characteristics of NESH have been investigated for over a decade, all earlier experiments were performed on non-neuronal cells. Therefore, the significance of NESH in neurons remains to be established.

In the current study, the characteristics of NESH were investigated in hippocampal neurons that undergo extensive actin cytoskeleton remodeling upon synaptic activity and play an important role in long-term memory. Our experiments revealed that the localization of NESH in dendrites is dynamically altered in an F-actin-dependent manner. The dynamics of NESH was additionally analyzed using the FRAP (fluorescence recovery after photobleaching) assay, and compared with that of GFP-tagged actin or postsynaptic scaffold proteins. Moreover, the effects of F-actin stabilization or disruption on molecular dynamics were evaluated via FRAP analysis. Remarkably, the F-actin-binding region of NESH appeared important for the regulation of NESH mobility. Moreover, induction of chemical LTP (long-term potentiation) led to synaptic translocation of NESH, and disruption of the F-actin cytoskeleton blocked the synaptic accumulation of NESH during LTP. Our results provide evidence to support the significance of the F-actin cytoskeleton in the dynamics and synaptic translocation of NESH in dendritic spines.

## Results

### NESH translocates to the synapse in an F-actin-dependent manner in hippocampal neurons

**Figure 1:**
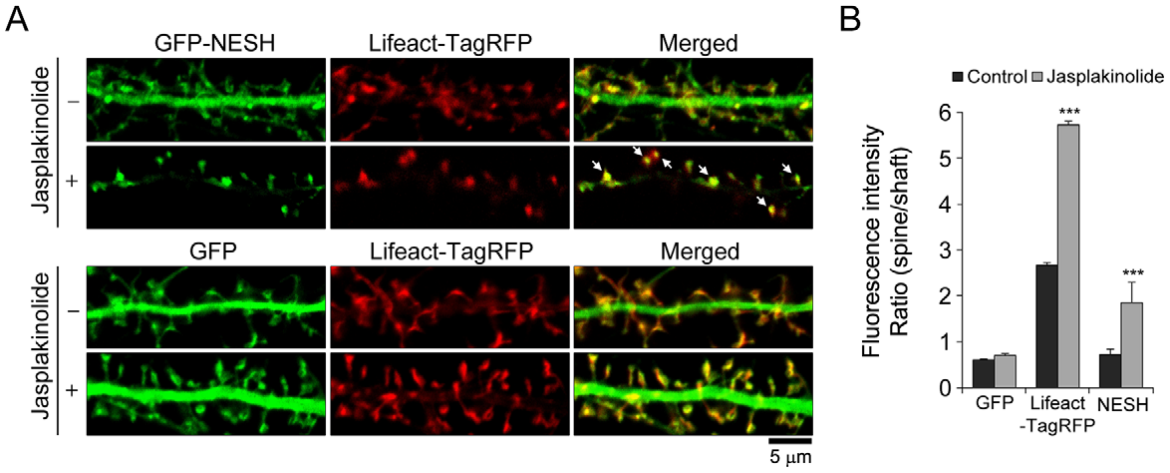
F-actin-dependent synaptic translocation of NESH in hippocampal neurons. (A) The effect of F-actin stabilization on localization of NESH was investigated. Hippocampal neurons were co-transfected with GFP-NESH (or GFP) and pLifeact-TagRFP at 10–12 DIV. pLifeact-TagRFP was employed to image the F-actin cytoskeleton within cells. Transfected neurons at 16–18 DIV were treated with jasplakinolide (5 µM for 10 min), fixed, and imaged. Colocalization between NESH and F-actin is indicated with white arrows in the merged image. (B) Quantitative analysis of the intensity ratio of spines vs. shafts from data obtained in Fig. 1A (N = 16 neurons for each condition). Data are presented as means ± SEM. ***p<0.001.

Previously, we found that NESH interacts with F-actin (filamentous actin) (unpublished data). F-actin is predominantly formed in dendritic spines and plays an important role in maintaining spine structure and anchoring PSD proteins at the synapse. Accordingly, we hypothesized that the integrity of the F-actin cytoskeleton is crucial for NESH localization in hippocampal neurons. To verify this hypothesis, NESH localization was examined in hippocampal neurons co-transfected with GFP-tagged NESH and pLifeact-TagRFP. The F-actin cytoskeleton was imaged using the Lifeact-TagRFP probe, which was selected on the basis of its lack of effects on actin polymerization and depolymerization within cells [16]. Under normal conditions, NESH was evenly localized throughout dendrites, including the shaft and spine regions (Fig. 1A). GFP, the control protein, diffused freely throughout dendrites. Lifeact-TagRFP was predominantly localized in the dendritic spine, but barely observed in dendritic shafts. To determine the effects of F-actin stabilization on NESH localization in hippocampal neurons, transfected neurons were treated with jasplakinolide, an F-actin-stabilizing reagent, and localization of NESH examined. Interestingly, localization of NESH was significantly altered after jasplakinolide treatment, displaying a marked increase in synaptic translocation into the dendritic spine. In addition, NESH localization overlapped with that of F-actin in the dendritic spine. Colocalization is indicated with white arrows in the merged image. On the other hand, GFP was not affected by jasplakinolide, and displayed similar localization to that observed under non-treated conditions. To quantitatively assess the synaptic translocation of NESH, spine vs. shaft intensity ratios were analyzed (Fig. 1B). As shown in Figure 1B, the ratio of F-actin (spine vs. shaft) was greatly increased after treatment with jasplakinolide, compared with normal conditions. The ratio of NESH intensity in spine was also markedly increased in jasplakinolide-treated neurons whereas the GFP intensity in spine was unchanged. The specificity of anti-NESH antibody was confirmed by immunoblotting and immunostaining (Fig. S1A, B). pLifeact-TagRFP-transfected neurons were treated with jasplakinolide for 10 min and then stained with anti-NESH antibody (Fig. S2A). As the result, the spine vs. shaft ratio of endogenous NESH was significantly increased in jasplakinolide-treated neurons (Fig. S2B). These data suggest that the stabilization of F-actin cytoskeleton leads to synaptic targeting of NESH.

### FRAP analysis of NESH dynamics within a single spine

**Figure 2:**
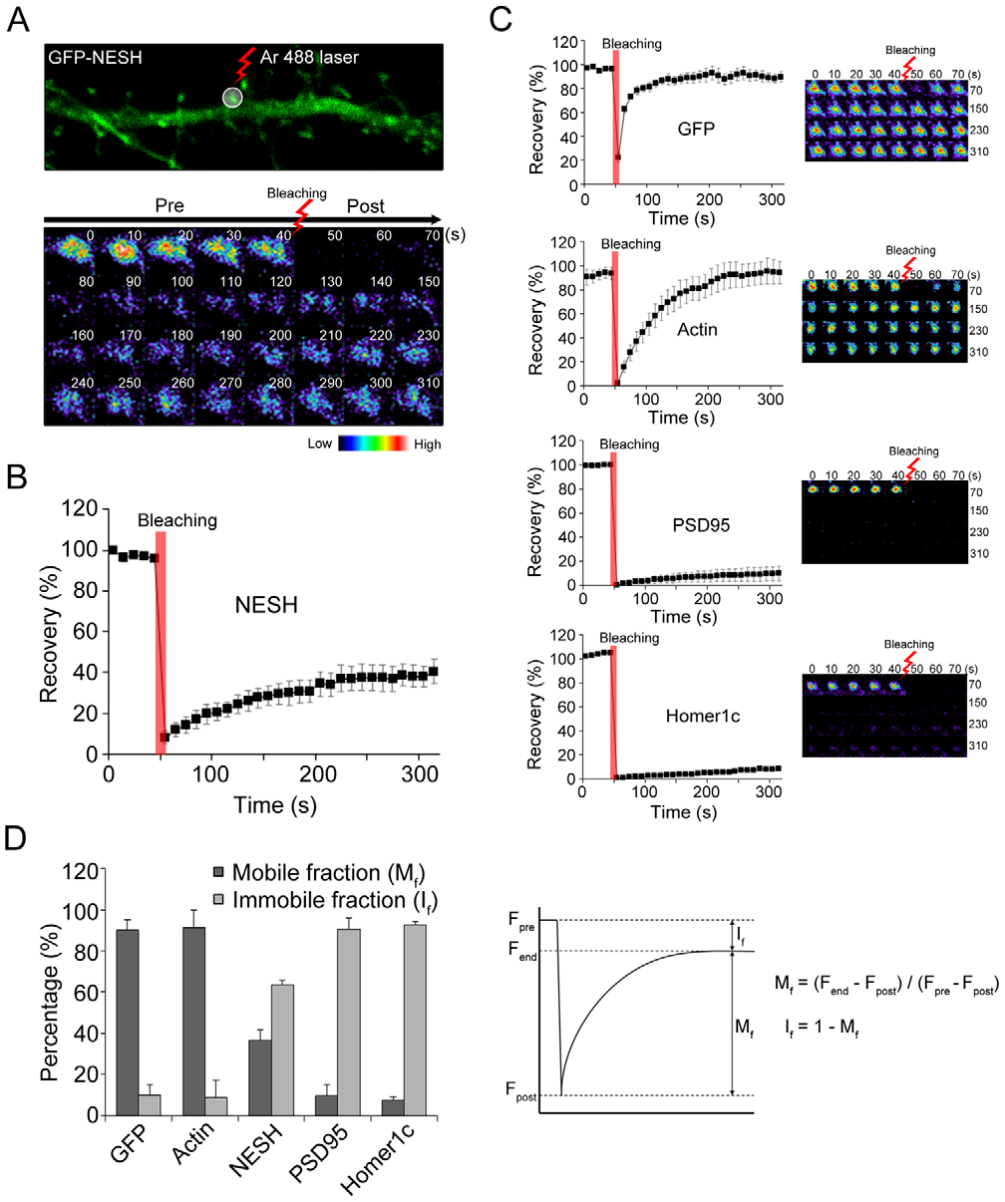
FRAP analysis of NESH dynamics in dendritic spines. (A) To investigate the dynamics of NESH in a single spine, the FRAP (fluorescence recovery after photobleaching) assay was performed. Hippocampal neurons at 10–12 DIV were transfected with GFP-NESH and subjected to FRAP imaging at 16–18 DIV. A single spine of GFP-NESH-transfected neurons was set to ROI (region of interest) and bleached for 7 s with an Ar 488 laser, and recovery of GFP-NESH observed at intervals of 10 s over a time-course of 5 min. (B) Recovery curve of GFP-NESH from data obtained in Fig. 2A. NESH fluorescence was slowly recovered (up to ∼40%) for 5 min (N = 15, data from three to five independent experiments). (C) Hippocampal neurons at 10–12 DIV were transfected with GFP, GFP-actin, GFP-PSD95 or GFP-Homer1c, and used for FRAP imaging at 16–18 DIV. NESH mobility was compared with that of other proteins using FRAP (N = 15 for each protein, data from three to five independent experiments). (D) Analysis of mobile/immobile fractions from data obtained in Fig. 2B and C. F_end_: fluorescence at the end time-point, F_post_: fluorescence right after photobleaching, F_pre_: fluorescence before photobleaching, M_f_: mobile fraction, I_f_: immobile fraction. Data are presented as means ± SEM.

FRAP analysis was conducted on a single dendritic spine, with a view to investigating the dynamics of NESH in detail. To analyze the movement of GFP-NESH, a circular region containing the spine was selected and photobleached with a laser, and the recovery of GFP-NESH fluorescence recorded at intervals of 10 s for 5 min in a time-course experiment (Fig. 2A). The fluorescence intensity of NESH was consistently maintained before bleaching, and fluorescence disappeared completely after bleaching for 7 s. Subsequently, fluorescence intensity was slowly recovered (up to about 40%) over 5 min (36.6±5.1% at 310 s), indicative of an exchangeable portion of NESH (Fig. 2B). To compare NESH dynamics with other proteins, FRAP analyses were conducted after transfection of GFP, GFP-actin, GFP-PSD95 and GFP-Homer1c. The fluorescence of GFP was fully recovered within 50 s after bleaching, which may be attributable to the fact that this protein is freely diffusing (90.1±5.2% at 310 s) (Fig. 2C, D). The actin cytoskeleton shows a very high turnover rate in dendritic spines. Thus, recovery of GFP-actin was rapidly completed within 5 min (91.4±8.5% at 310 s). In contrast, the postsynaptic scaffold proteins, PSD95 and Homer1c showed very low recovery rates (9.4±5.5% at 310 s for GFP-PSD95, 7.4±1.8% at 310 s for GFP-Homer1c), indicating that these proteins are relatively immobile and attached to the postsynaptic density with high affinity (Fig. 2C, D).

### NESH mobility is affected by F-actin stabilization in the dendritic spine

**Figure 3:**
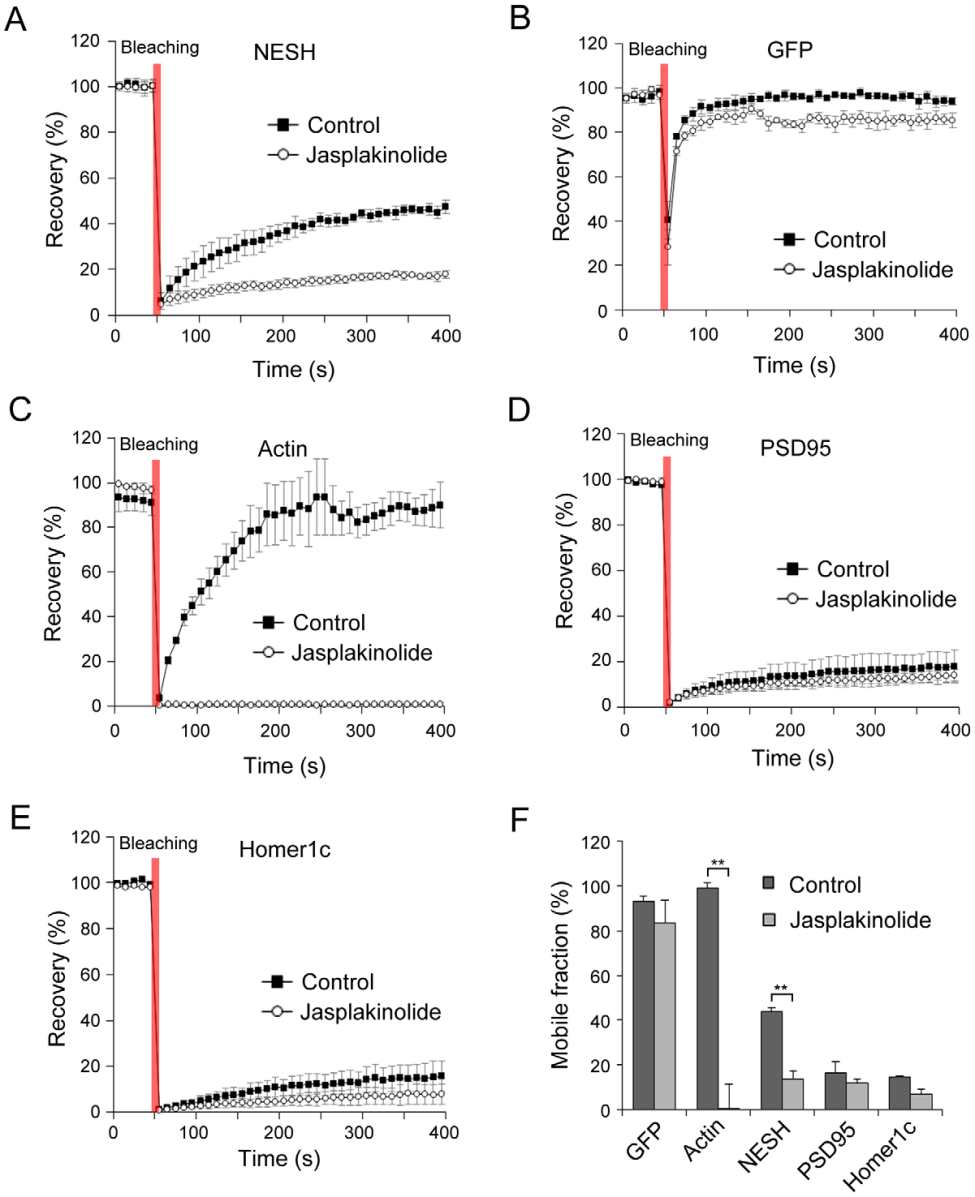
Effect of F-actin stabilization on NESH mobility in dendritic spines. FRAP analysis was performed to examine the effect of F-actin stabilization on NESH mobility. Hippocampal neurons at 10–12 DIV were transfected with GFP-NESH, GFP, GFP-actin, GFP-PSD95 or GFP-Homer1c, and subjected to FRAP analysis at 16–18 DIV. (A, F) Recovery curves of GFP-NESH in non-treated (control) and jasplakinolide-treated neurons (mobile fractions: 43.9±2.4% at 400 s for control, 13.6±2.8% at 400 s for jasplakinolide). (B, F) Recovery curves of GFP (mobile fractions: 92.7±8.0% at 400 s for control, 83.3±1.3% at 400 s for jasplakinolide). (C, F) Recovery curves of GFP-actin (mobile fractions: 98.6±8.7% at 400 s for control, 0.2±0.1% at 400 s for jasplakinolide). (D–F) Recovery curves and mobile fractions of the scaffolding proteins, PSD95 and Homer1c (control: 16.7±5.2% at 400 s for GFP-PSD95, 14.8±6.6% at 400 s for GFP-Homer1c, jasplakinolide: 12.0±2.7% at 400 s for GFP-PSD95, 7.0±4.2% at 400 s for GFP-Homer1c). (F) Analysis of the mobile/immobile fractions from data obtained in Fig. 3A–E (N = 15 for each condition, data from three to five independent experiments). Data are presented as means ± SEM. **p<0.01.

Localization of NESH was affected by F-actin stabilization in Figure 1, showing that the protein is predominantly accumulated in dendritic spines. To examine the change in NESH mobility after F-actin stabilization, hippocampal neurons were treated with jasplakinolide and analyzed using the FRAP assay. The mobile fraction of NESH was significantly reduced in jasplakinolide-treated neurons (13.6±2.8% at 400 s), compared with non-treated neurons (43.9±2.4% at 400 s) (Fig. 3A, F). In jasplakinolide-treated neurons, the mobile fraction of NESH was similar to that of the PSD scaffold proteins, PSD95 and Homer1c. The data suggest that stabilization of the F-actin cytoskeleton alters the dynamics of NESH and immobilizes it at synapses, possibly as a result of increased interactions with F-actin. Converse results were obtained with GFP and GFP-actin in FRAP analyses. The recovery of GFP was not significantly affected by jasplakinolide (92.7±8.0% at 400 s for control, 83.3±1.3% at 400 s for jasplakinolide) (Fig. 3B, F). On the other hand, actin recovery was completely blocked after F-actin stabilization, indicating near-complete suppression of actin dynamics (98.6±8.7% at 400 s for control, 0.2±0.1% at 400 s for jasplakinolide) (Fig. 3C, F). The recovery levels of PSD95 and Homer1c were not significantly altered in the presence of jasplakinolide (12.0±2.7% at 400 s for GFP-PSD95, 7.0±4.2% at 400 s for GFP-Homer1c), since the levels of recovery in non-treated neurons (control) were initially very low at ∼10% (Fig. 3D–F).

### NESH mobility is affected by disruption of F-actin in dendritic spines

**Figure 4:**
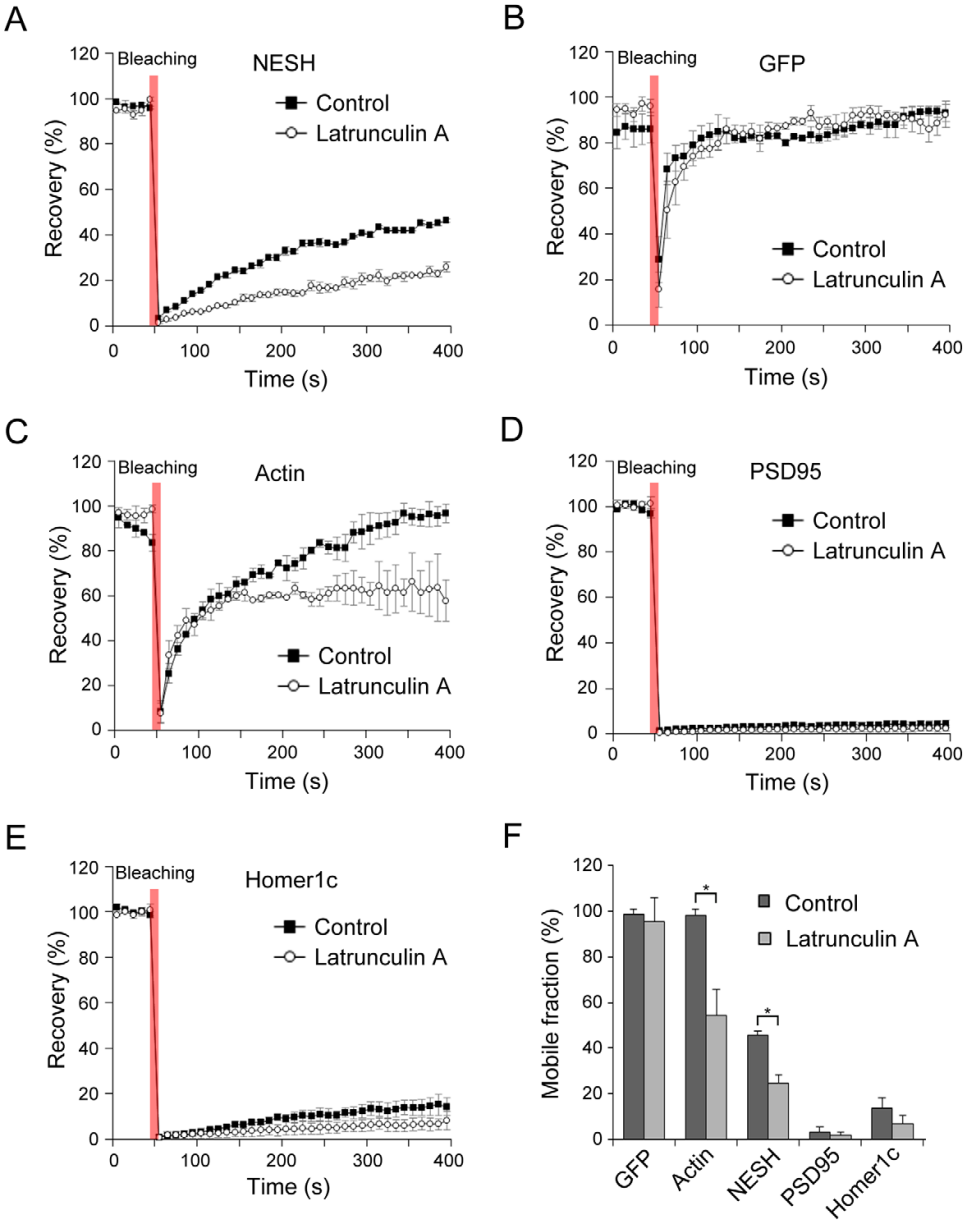
Effect of F-actin disruption on mobility of NESH. To examine the effect of F-actin disruption on NESH mobility in the dendritic spine, FRAP analyses were performed using hippocampal neurons at 16–18 DIV that were transfected with GFP-NESH, GFP, GFP-actin, GFP-PSD95 and GFP-Homer1c at 10–12 DIV. F-actin disruption was induced by treating with latrunculin A (5 µM for 10 min). (A, F) Recovery curves of GFP-NESH in non-treated (control) and latrunculin A-treated neurons (mobile fractions: 45.6±1.9% at 400 s for control, 24.9±3.6% at 400 s for latrunculin A), suggesting that dynamic actin remodeling is crucial for the mobility and dynamics of NESH. (B, F) Recovery curves of GFP (mobile fractions: 98.2±2.2% at 400 s for control, 95.4±10.3% at 400 s for latrunculin A) (C, F) Recovery curves of GFP-actin (mobile fractions: 98.1±2.6% at 400 s for control, 54.2±11.4% at 400 s for latrunculin A) (D–F) Recovery curves and mobile fractions of scaffold proteins, GFP-PSD95 and GFP-Homer1c, showed no significant differences. (F) Analysis of mobile/immobile fractions from data obtained in Fig. 4A–E (N = 15 for each condition, data from three to five independent experiments). Data are presented as means ± SEM. *p<0.05.

Stabilization of actin filaments affects the localization and dynamics of NESH, indicating close linkage of this protein with the F-actin cytoskeleton. Accordingly, we investigated whether NESH dynamics is affected by F-actin disruption. Hippocampal neurons were transfected with GFP-NESH and treated with latrunculin A to induce actin depolymerization. Subsequently, NESH mobility was analyzed with FRAP. Disruption of actin filaments by latrunculin A led to a significant reduction in the mobile fraction of NESH, compared with that in non-treated neurons (control) (45.6±1.9% at 400 s for control, 24.9±3.6% at 400 s for latrunculin A) (Fig. 4A, F). These data suggest that the integrity of the F-actin cytoskeleton and dynamic actin remodeling are essential for the mobility and dynamics of NESH. Similar to jasplakinolide-treated neurons, latrunculin A treatment did not affect the recovery of free-diffusing GFP (98.2±2.2% at 400 s for control, 95.4±10.3% at 400 s for latrunculin A) (Fig. 4B, F). In particular, recovery of GFP-actin was severely affected by latrunculin A (98.1±2.6% at 400 s for control, 54.2±11.4% at 400 s for latrunculin A), implying that actin polymerization in spine is crucial for dynamic actin turnover (Fig. 4C, F). In the case of GFP-PSD95 and GFP-Homer1c, recovery was not altered by F-actin disruption, indicating that the motilities of these proteins are independent of the F-actin cytoskeleton (Fig. 4D–F).

### The F-actin-binding region of NESH is crucial for dynamics

**Figure 5:**
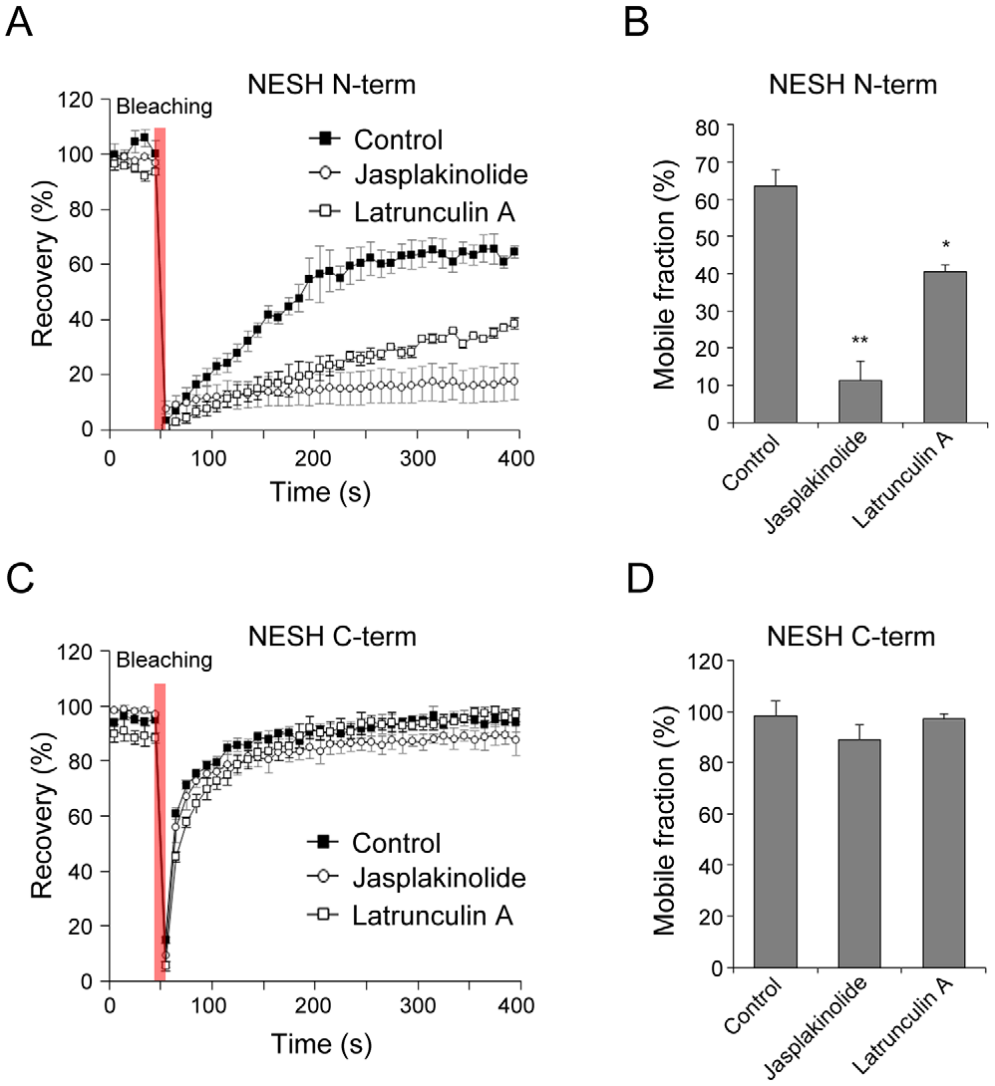
Importance of the F-actin-binding region in NESH dynamics. The dynamics of NESH-N-term (N-terminal half of NESH, F-actin binding region) and C-term (C-terminal half of NESH, F-actin non-binding region) were analyzed using FRAP. Hippocampal neurons at 10–12 DIV were transfected with GFP-NESH-N-term or GFP-NESH-C-term, and subjected to FRAP analysis at 16–18 DIV. (A, B) Recovery curves and mobile fractions of NESH-N-term (63.3±4.5% at 400 s for control, 11.2±5.1% for jasplakinolide, 40.4±1.7% for latrunculin A). N = 15 for each condition, data from three to five independent experiments. (C, D) Recovery curves and mobile fractions of NESH-C-term (98.1±6.0% for control, 89.0±5.7% at 400 s for jasplakinolide, 97.0±2.0% at 400 s for latrunculin A). N = 15 for each condition, data from three to five independent experiments. Data are presented as means ± SEM. *p<0.05, **p<0.01.

Earlier experiments showed that the dynamics of NESH in dendritic spine is affected by both F-actin stabilization and disruption induced by jasplakinolide and latrunculin A. This finding implies that actin dynamics is fundamental for the mobility of NESH. The N-terminal half of NESH interacts solely with F-actin (unpublished data). Accordingly, we examined whether the F-actin binding capacity is important for NESH mobility. Hippocampal neurons were transfected with GFP-NESH-N-term (N-terminal half, amino acids 1–229) or GFP-NESH-C-term (C-terminal half, amino acids 221–367), followed by FRAP analyses. The recovery of NESH N-term was 63.3±4.5% at 400 s under normal conditions (Fig. 5A, B), while that of NESH C-term was 98.1±6.0% at 400 s (Fig. 5C, D). Interestingly, the recovery curve of NESH C-term was similar to that of free-diffusing GFP, indicating that this region of the protein does not make any associations in PSD, even with the actin cytoskeleton. Consistent with this theory, the recovery curve of NESH C-term was not significantly altered after treatment with jasplakinolide or latrunculin A (89.0±5.7% at 400 s for jasplakinolide, 97.0±2.0% at 400 s for latrunculin A), confirming no association with the actin cytoskeleton (Fig. 5C, D). In contrast, the mobility of NESH N-term was severely affected by F-actin-stabilizing and -disrupting reagents. F-actin stabilization significantly lowered the mobile fraction of NESH N-term to 11.2±5.1% at 400 s. Moreover, the recovery of NESH N-term was significantly reduced in latrunculin A-treated neurons (40.4±1.7% at 400 s) (Fig. 5A, B). Our findings clearly suggest that the F-actin binding capacity of NESH is critical for its dynamics in hippocampal neurons.

### NESH translocates into dendritic spines via chemical LTP induction

**Figure 6:**
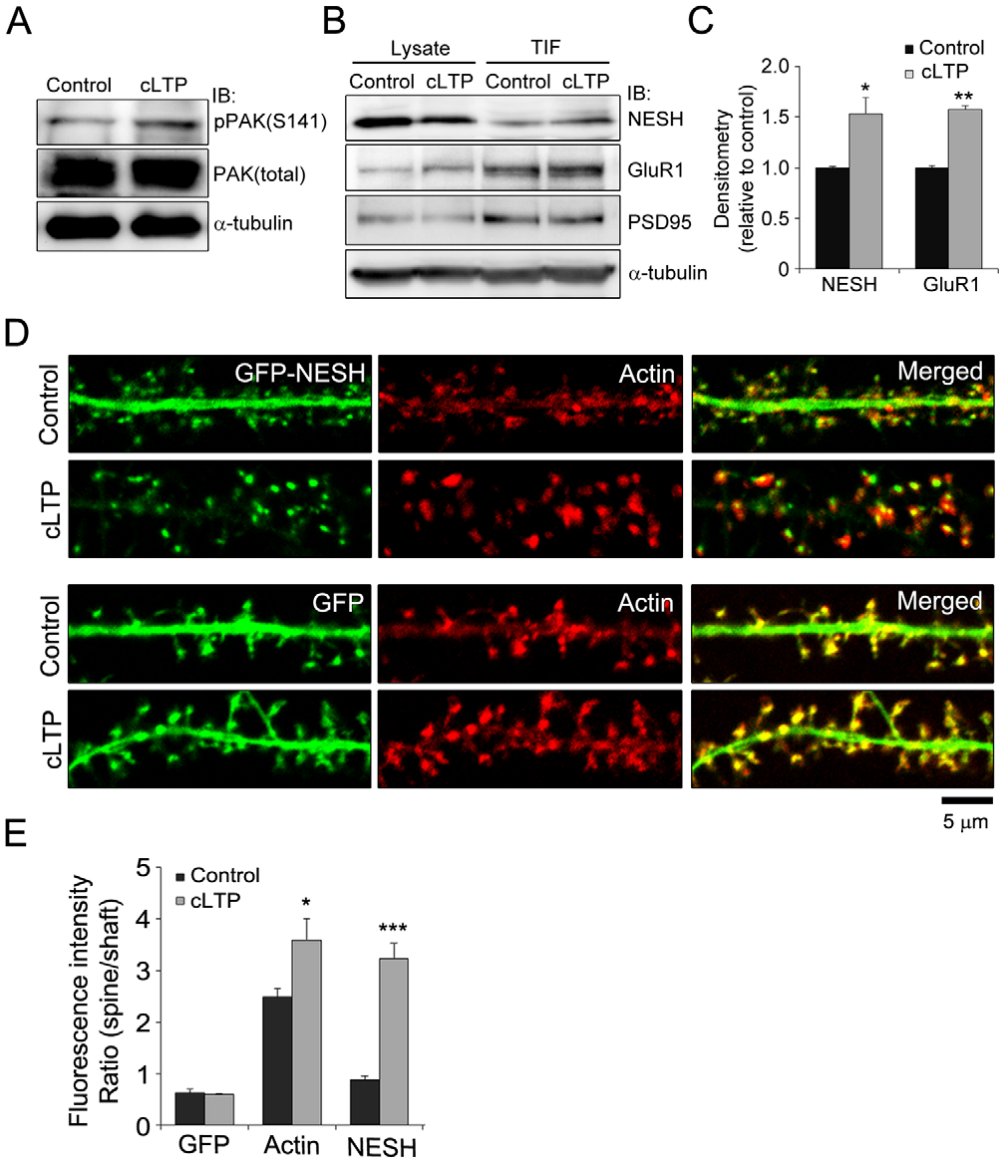
Synaptic translocation of NESH via LTP induction. The glycine-induced chemical LTP (long-term potentiation) method was used to mimic physiological LTP. (A) Chemical LTP (cLTP) was induced in hippocampal neurons at 16–18 DIV, and the level of phospho-PAK assessed to ascertain cLTP induction. (B) After cLTP induction, the Triton X-100-insoluble fraction was extracted from hippocampal neurons, and the association between NESH and cytoskeleton or synaptic site examined using immunoblot analysis. (C) The band intensities were quantified and normalized by loading control (α-tubulin). (D) Synaptic translocation of NESH was examined during LTP. Hippocampal neurons at 10–12 DIV were transfected with GFP-NESH (or GFP). After cLTP induction at 16–18 DIV, transfected neurons were fixed and stained with Alexa 594-conjugated phalloidin, and NESH localization examined. GFP served as the control. (E) Analysis of the fluorescence intensity ratio in dendritic spine vs. shaft from data obtained in Fig. 6D (N = 12–16 neurons for each condition). Data are presented as means ± SEM. *p<0.05, **p<0.01, ***p<0.001.

The F-actin content in dendritic spines is increased during LTP [17]. The F-actin cytoskeleton serves in the trafficking of AMPA receptors into the postsynaptic region while also acting as a scaffold for plasticity proteins. In addition, the actin cytoskeleton participates in the enlargement and maintenance of spine volume, thereby aiding in the maintenance of LTP [7], [18], [19], [20]. Since NESH displays F-actin-dependent movement in the dendritic spine, we investigated the effects of F-actin increase during LTP on NESH dynamics. To mimic physiological LTP, the glycine-induced chemical LTP (cLTP) method was used, since it specifically stimulates NMDA receptors only at synapses receiving spontaneous release of glutamate, thus reproducing stimulus-induced synaptic potentiation [21]. First, the level of phospho-PAK (S141) was assessed, in view of the finding that PAK is activated after cLTP induction [21]. Our results showed a significant increase in the phospho-PAK level in cLTP (Fig. 6A). Cytoskeletal elements and postsynaptic density (PSD) are resistant to the nonionic detergent, Triton X-100. To investigate the association between NESH and the cytoskeleton or synaptic site, hippocampal neurons were treated with Triton X-100, and the soluble fraction removed. Purified anti-NESH antibody was used to detect endogenous NESH (Fig. S1). NESH was more enriched in TIF (Triton X-100 insoluble fraction) after cLTP induction, and the level of GluR1, an AMPA receptor subunit, was increased in TIF (Fig. 6B, C). These data suggest that NESH is more strongly associated with cytoskeletal elements or synaptic sites during LTP. To clarify the association of NESH with synaptic sites during LTP, hippocampal neurons were transfected with GFP-NESH, followed by staining with Alexa 594-conjugated phalloidin, and localization of NESH examined (Fig. 6D). GFP served as the control. The fluorescence intensity ratio of spine vs. shaft was measured to establish synaptic association (or translocation) (Fig. 6E). The GFP ratio was not changed after cLTP induction. In contrast, the F-actin ratio (spine vs. shaft) was increased. Interestingly, NESH additionally translocated into the synapse after cLTP induction. Quantitative analysis further revealed a 3-fold increase in the ratio of NESH intensity (spine vs. shaft) after cLTP induction, compared with control. Synaptic translocation of endogenous NESH was also examined during cLTP induction. Hippocampal neurons were transfected with pLifeact-TagRFP to visualize F-actin cytoskeleton. As the result, the ratio of NESH intensity (spine vs. shaft) was markedly increased after cLTP induction (Fig. S2C, D).

### Synaptic translocation of NESH during chemical LTP is dependent on the F-actin cytoskeleton

**Figure 7:**
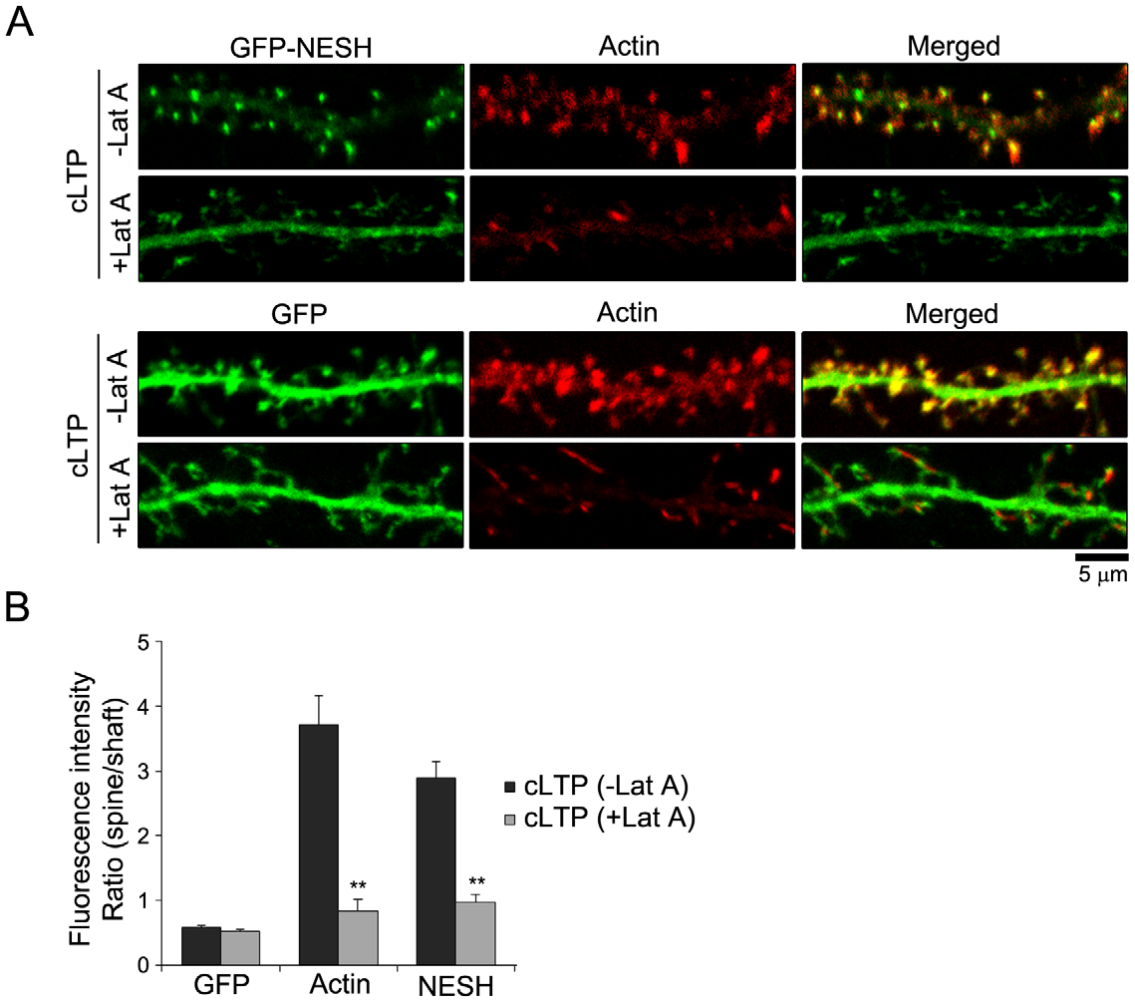
F-actin-dependent synaptic translocation of NESH after LTP induction. (A) Hippocampal neurons at 10–12 DIV were transfected with GFP-NESH (or GFP). To determine the importance of the F-actin cytoskeleton in NESH translocation during cLTP, transfected neurons at 16–18 DIV were treated with latrunculin A (5 µM for 10 min), and cLTP was subsequently induced. Following fixing of neurons, NESH localization was examined. (B) Analysis of the intensity ratio (spine vs. shaft) from data obtained in Fig. 7A (N = 11–13 neurons for each condition). Data are presented as means ± SEM. **p<0.01.

To determine the importance of the F-actin cytoskeleton in the synaptic translocation of NESH during cLTP, hippocampal neurons were treated with latrunculin A for 10 min, followed by induction of cLTP. Subsequently, neurons were fixed, and localization of NESH examined. As shown in Figure 7A, latrunculin A induced complete removal of the F-actin cytoskeleton from dendritic spines. Moreover, NESH translocation was completely blocked by latrunculin A, even though cLTP was induced. Analysis of the spine vs. shaft ratio confirmed this result (Fig. 7B). However, the GFP intensity ratio was not significantly affected by actin depolymerization induced by latrunculin A (Fig. 7A, B). Based on these results, we propose that the F-actin cytoskeleton is crucial for NESH translocation into the synapse during cLTP.

## Discussion

In response to synaptic stimuli, postsynaptic proteins are reorganized via translocation [22], [23]. Here, we have shown that localization of NESH is regulated by synaptic activity. In our experiments, NESH was evenly distributed throughout dendrites, including shafts and spines, in the resting state. However, cLTP induction led to translocation of NESH from dendritic shafts to postsynaptic sites. Actin, the major cytoskeleton constituent in the dendritic spine is very dynamic, continuously transforming between G-actin (globular monomeric actin) and F-actin (filamentous actin) states according to synaptic stimuli. LTP induction shifts the G-actin/F-actin ratio in favor of F-actin [24]. NESH specifically binds to F-actin, but not G-actin (unpublished data). Accordingly, we speculated that synaptic translocation of NESH is related to the increased F-actin content at synapses during LTP. As expected, synaptic accumulation of NESH was blocked in latrunculin A-treated neurons, even though LTP was induced, suggesting that synaptic targeting of NESH is closely related to actin cytoskeleton remodeling. This theory is supported by several earlier reports that emphasize the link between NESH and the actin cytoskeleton. NESH is reported to interact with IRSp53, an actin regulator, and is involved in PDGF-induced membrane ruffling, a dynamic F-actin-rich structure [15]. Additionally, NESH is a member of the WAVE complex, an actin regulator, and interacts with PAK (p21-activated kinase), a Rac/Cdc42 effector, thus providing a connection with the actin cytoskeleton [13], [14]. We confirmed these associations using F-actin-stabilizing reagents. Stabilization of F-actin filaments by jasplakinolide induced synaptic translocation of NESH from the shaft to spine, compared with non-stimulated conditions. These findings collectively support the importance of the F-actin cytoskeleton in determining the localization and synaptic translocation of NESH.

Reorganization and dynamic remodeling of PSD is believed to be critical in postsynaptic signal transmission [25], [26]. PSD scaffolding proteins interact with multiple signaling molecules, including kinases and phosphatases. Thus, changes in scaffold proteins in PSD reflect changes in postsynaptic signaling, indicating regulation of signal transduction via remodeling of PSD. In addition, a variety of PSD scaffold proteins interact with the actin cytoskeleton, and the intact actin meshwork is reported as crucial in the maintenance and remodeling of the PSD composition [27], [28]. PSD proteins display distinct dynamics, based on either differential intermolecular interactions of scaffolding proteins or variable dependencies on the actin cytoskeleton [29]. FRAP analysis revealed that the mobile fraction of NESH is about 40% at 5 min after photobleaching. We assume that the mobile fraction is weakly attached in the PSD site or associated with dynamic cytoskeleton components. In relation to actin and PSD scaffolding proteins, mobile fractions were in the following order: actin ≫ NESH > Homer1c = PSD95. Fluorescence of actin in the dendritic spine was fully recovered within a short time, showing a high turnover rate, while scaffold proteins PSD95 and Homer1c were recovered to below 15% within 5 min after photobleaching, implying that the proteins are tightly attached in PSD and less dynamic. Since actin dynamics is involved in remodeling of the PSD molecular composition, the molecular dynamics of NESH was measured after pharmacological stabilization or disruption of the F-actin cytoskeleton. In FRAP analysis, the turnover rate of NESH was significantly affected by F-actin stabilization, since the protein became more immobile. Our data provide strong evidence that the actin cytoskeleton is closely involved in NESH dynamics. The F-actin cytoskeleton may act as a platform for anchoring PSD proteins within the spine. Therefore, it is likely that stabilized actin filaments provide more static platforms for their binding molecules, leading to decreased molecular dynamics. We assume that this theory is also applicable to NESH. PSD95 and Homer1c are associated with F-actin [30], [31]. Accordingly, we expected these proteins to be affected by F-actin stabilization. However, it was difficult to observe these effects in FRAP analysis, since the basal levels of the mobile fractions of these proteins were too low. Treatment with latrunculin A sequesters monomeric G-actin and blocks the formation of F-actin content in dendritic spine. FRAP analysis revealed a significant decrease in the recovery of NESH in F-actin-deficient spine. We assume that the ability of F-actin to act as a platform for anchoring PSD molecules is reduced as a result of disruption at the postsynapse, resulting in decreased recovery of NESH in synapse.

In a previous report, it has shown that an F-actin-binding domain is necessary for efficient synaptic targeting [23]. The F-actin-binding region of NESH was found to be crucial for synaptic targeting in FRAP analysis. The N-terminal half of NESH specifically, the F-actin-binding region, was affected by F-actin stabilization and disruption, which induced diminished fluorescence recovery after photobleaching. On the other hand, the C-terminal half of NESH showed a similar recovery pattern to free-diffusing GFP protein. Jasplakinolide and latrunculin A treatment had no effect on NESH C-term-transfected neurons, indicating that the C-terminal half of NESH does not associate at postsynapse. These data clearly indicate that the F-actin cytoskeleton is essential for synaptic targeting and dynamics of NESH.

In summary, we have uncovered the regulatory mechanism underlying synaptic targeting of NESH from the dendritic shaft to spine in hippocampal neurons. Our findings support an essential role of the F-actin cytoskeleton in synaptic targeting and dynamic mobility of NESH in PSD. It would be interesting to further elucidate the functional role of NESH in PSD remodeling by synaptic activity, based on the F-actin-dependent regulation of its mobility and synaptic translocation.

## Materials and Methods

### Ethics Statement

All animal experiments were approved by the Gwangju Institute of Science and Technology Animal Care and Use Committee (the permit number: GIST-2008-36).

### Antibodies and reagents

Rabbit polyclonal anti-NESH antibody was generated using the C-terminal region of NESH (amino acids 204–367). cDNA corresponding to the C-terminal region was amplified by PCR and subcloned into pGEX4T-1 vector for GST fusion protein expression. The GST fusion protein was purified according to the manufacturer's protocol and used for immunization. After the fifth injection, serum specificity was tested via immunoblot analysis and further purified with affinity chromatography. Rabbit polyclonal anti-PAK antibody was purchased from Santa Cruz Biotechnology (CA, USA), and rabbit polyclonal anti-PAK1/2/3(pS141) from Invitrogen. Mouse monoclonal anti-α-tubulin antibody was purchased from Sigma, rabbit polyclonal anti-GluR1 antibody from Calbiochem, and mouse monoclonal anti-PSD95 antibody from Abcam. Alexa Fluor 594-conjugated phalloidin was acquired from Molecular probes (Eugene, OR, USA). Horseradish peroxidase (HRP)-conjugated anti-mouse or -rabbit secondary antibodies were purchased from The Jackson Laboratory. Jasplakinolide A and latrunculin A were acquired from Molecular Probes, bicuculline methiodide, tetrodotoxin (TTx) and strychnine hydrochloride from Tocris, and Glycine from Sigma.

### Plasmids

cDNA encoding full-length NESH was amplified by PCR from a rat brain cDNA library and subcloned into pEGFP-C2 vector (BD Clontech, Palo Alto, CA). The NESH N-terminal (N-term, amino acids 1–229) and C-terminal (C-term, amino acids 221–369) halves were subcloned into pEGFP-C2 vector. The pLifeact-TagRFP construct was purchased from ibidi (Germany). GFP-PSD95 and GFP-Homer1c were kindly provided by Dr. Okabe (Tokyo Medical and Dental University, Japan), and the GFP-β-actin construct by Dr. Beat Imhof (Centre Medical Universitaire, Geneva, Switzerland).

### Cell culture, transfection and immunocytochemistry

For primary neuronal cultures, hippocampal neurons were dissected from E18-E19 Sprague–Dawley rat embryos, dissociated with papain (Worthington Biochemical Corp., Lakewood, NJ, USA) and plated on poly-D-lysine-coated coverslips at a density of 3×10^5^ cells/60 mm plastic dish. Neuronal cultures were maintained in Neurobasal Medium (Invitrogen) supplemented with B-27 (Invitrogen) and 2 mM GlutaMAX (Invitrogen). Neurons were transfected using a modified calcium phosphate precipitation method. Transfected neurons were fixed with 4% paraformaldehyde/4% sucrose in PBS and permeabilized with 0.1% Triton X-100. F-actin was stained with Alexa Fluor 594-conjugated phalloidin for 30 min at 37°C in specific experiments. Images were acquired with a FV1000 confocal microscope (Olympus, Tokyo, Japan).

### Preparation of Triton X-100 insoluble fraction and immunoblot analysis

Triton X-100 insoluble fraction was prepared as described previously [23]. Briefly, hippocampal neurons were extracted with Triton X-100 buffer (10 mM PIPES [pH 6.8], 0.5% Triton X-100, 50 mM NaCl, 3 mM MgCl_2_ and 300 mM sucrose) for 10 min on ice and washed with PBS containing 1 mM CaCl_2_, and 1 mM MgCl_2_. The Triton X-100 insoluble fraction was collected by adding SDS boiling lysis buffer, and subjected to subsequent SDS-PAGE and immunoblot analysis. For immunoblotting, the protein concentration in lysates was determined using the BCA protein assay (Pierce, Rockford, IL). SDS-PAGE was performed using 8% and 10% polyacrylamide gels, which were transferred to polyvinylidene fluoride (PVDF) membranes. After blocking with 5% nonfat dry milk or 3% BSA, membranes were incubated with the primary antibody. Positive bands were detected using HRP-coupled secondary antibodies and visualized using enhanced chemiluminescence (ECL).

### Induction of chemical LTP

Chemical LTP was induced as described previously [21]. Briefly, hippocampal neurons were maintained in normal ACSF (5 mM HEPES [pH 7.3], 125 mM NaCl, 2.5 mM KCl, 2 mM CaCl_2_, 1 mM MgCl_2_ and 33 mM glucose). Osmolarity was adjusted to 290 mosmol/l. LTP was induced by changing the medium to ACSF (5 mM HEPES [pH 7.3], 125 mM NaCl, 2.5 mM KCl, 2 mM CaCl_2_, 33 mM glucose, 0.2 mM glycine, 0.02 mM bicuculline and 0.003 mM strychnine) for 10 min at room temperature After that, the incubation solution was altered back to normal ACSF.

### Live-cell imaging and FRAP (fluorescence recovery after photobleaching)

For live-cell imaging, the culture medium was replaced with Tyrode's solution (25 mM HEPES [pH 7.4], 119 mM NaCl, 2.5 mM KCl, 2 mM CaCl_2_, 2 mM MgCl_2_ and 30 mM glucose) and placed in a chamber for 30 min before the experiment. The chamber was maintained at 37°C by setting the temperature of the chamber body at 37°C, objective lens at 39°C and chamber lid at 40°C. The chamber was supplied with continuous humidified 5% CO_2_ to maintain medium pH. A concentrated stock of latrunculin A or jasplakinolide in pre-warmed Tyrode's solution was applied. The final concentrations of latrunculin A and jasplakinolide solutions were 5 µM. Images were obtained using a 100× oil-immersion lens equipped with a Fluoview FV1000 confocal laser-scanning microscope with additional zoom factor 3. FRAP experiments were performed using a macro function of the stimulus setting menu in Fluoview software to control sequential image acquisition and emission of a photobleaching laser pulse to the ROI (region of interest). A single dendritic spine of hippocampal neuron was set as ROI and five pre-bleaching images acquired at 10 s intervals, and the fluorescence of spine photobleached for 7 s with an Argon 488 laser. The recovery of fluorescence was traced for an additional 5 min by acquiring images at 10 s intervals. Minimum laser power was used to prevent photobleaching during the pre- and post-bleaching stages. Pre-bleaching, bleaching and post-bleaching images were utilized for analyzing the dynamics of target proteins.

### Image analysis

The average intensity values of ROI, total image and background fluorescence were obtained from FRAP images. Background values were subtracted from those of ROI and total images for all time-points. Subsequently, the recovery curve was plotted with the ROI value in relation to the total value over the time-course. Finally, the curve was normalized to the value of first pre-bleaching time-point, taken as 1. Based on the plot, mobile and immobile fractions were calculated to describe the kinetics of fluorescence. Specifically, mobile and immobile fractions were determined by calculating the ratios of the final to initial fluorescence intensity. Fluorescence intensities of the end time-point (F_end_), start time-point (F_pre_) and time-point after photobleaching (F_post_) were determined, and mobile (M_f_) and immobile (I_f_) fractions calculated using the following equations:
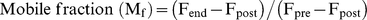


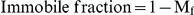



To examine the translocation of proteins into the dendritic spine, the spine/shaft fluorescence intensities were analyzed as the ratio of the average fluorescence intensities of spine to those in the adjacent dendritic shaft. Measurements were performed using MetaMorph imaging software (Universal Imaging Cooperation, West Chester, PA). Statistical significance was determined using the Student's t test.

## Supporting Information

Figure S1 **Generation of rabbit polyclonal anti-NESH antibody.** (A) Rabbit polyclonal anti-NESH antibody was generated using the C-terminal region of NESH (amino acids 204–367). Antibody specificity was tested with immunoblot analysis using whole brain lysate and further confirmed with the antibody purified using antigen-conjugated affinity chromatography. (B) To test specificity of anti-NESH antibody, hippocampal neurons were transfected with GFP or GFP-NESH and fixed, and then stained with anti-NESH antibody.
(TIF)

Figure S2 **Synaptic translocation of endogenous NESH by F-actin stabilization and cLTP induction.** (A) Hippocampal neurons were transfected with pLifeact-TagRFP at 10–12 DIV. pLifeact-TagRFP was used to visualize F-actin within cells. Transfected neurons at 16–18 DIV were treated with jasplakinolide (5 µM for 10 min), fixed, and stained with anti-NESH antibody. Colocalization between NESH and F-actin is indicated with white arrows in the merged image. (B) The intensity ratio (spine vs. shaft) was quantitatively analyzed from data obtained in Fig. S2A (N = 12 neurons for control, N = 19 neurons for jasplakinolide). (C) Synaptic translocation of endogenous NESH was examined during LTP. Hippocampal neurons at 10–12 DIV was transfected with pLifeact-TagRFP. After cLTP induction at 16–18 DIV, transfected neurons were fixed and stained with anti-NESH antibody, and NESH localization examined. White arrows in merged image indicate colocalization between NESH and F-actin. (D) Analysis of the fluorescence intensity ratio in dendritic spine vs. shaft from data obtained in Fig. S2C (N = 21 neurons for each condition). Data are presented as means ± SEM. *p<0.05, ***p<0.001.
(TIF)
