# Supplementary figures and images for: F-Actin-Dependent Regulation of NESH Dynamics in Rat Hippocampal Neurons
Source: PLoS One. 2012 Apr 4;7(4):e34514. doi: 10.1371/journal.pone.0034514 (PMC3319579; doi:10.1371/journal.pone.0034514)

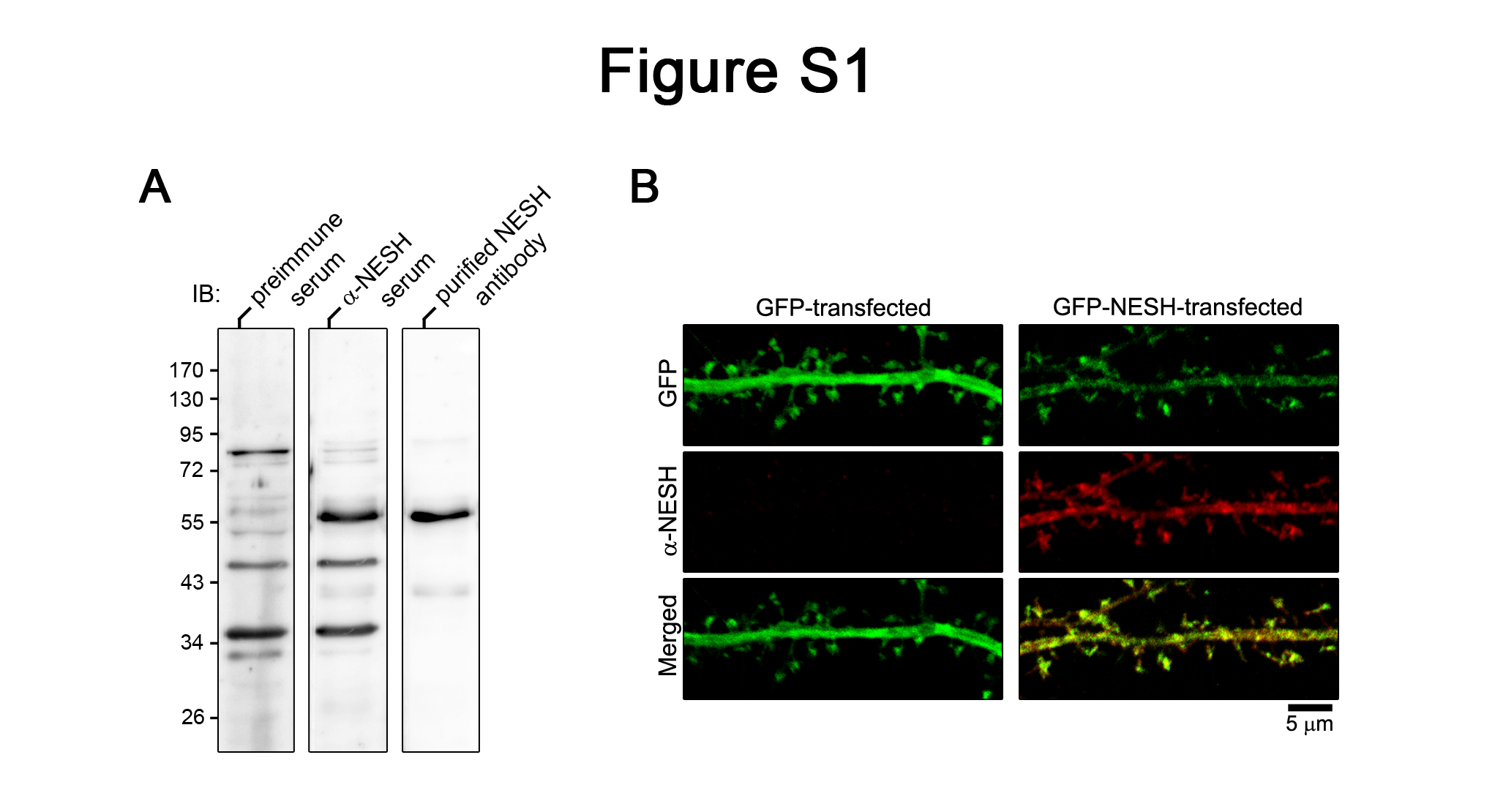

Supplement: Figure S1 — Generation of rabbit polyclonal anti-NESH antibody. (A) Rabbit polyclonal anti-NESH antibody was generated using the C-terminal region of NESH (amino acids 204–367). Antibody specificity was tested with immunoblot analysis using whole brain lysate and further confirmed with the antibody purified using antigen-conjugated affinity chromatography. (B) To test specificity of anti-NESH antibody, hippocampal neurons were transfected with GFP or GFP-NESH and fixed, and then stained with anti-NESH antibody. (TIF) [file pone.0034514.s001.tif]

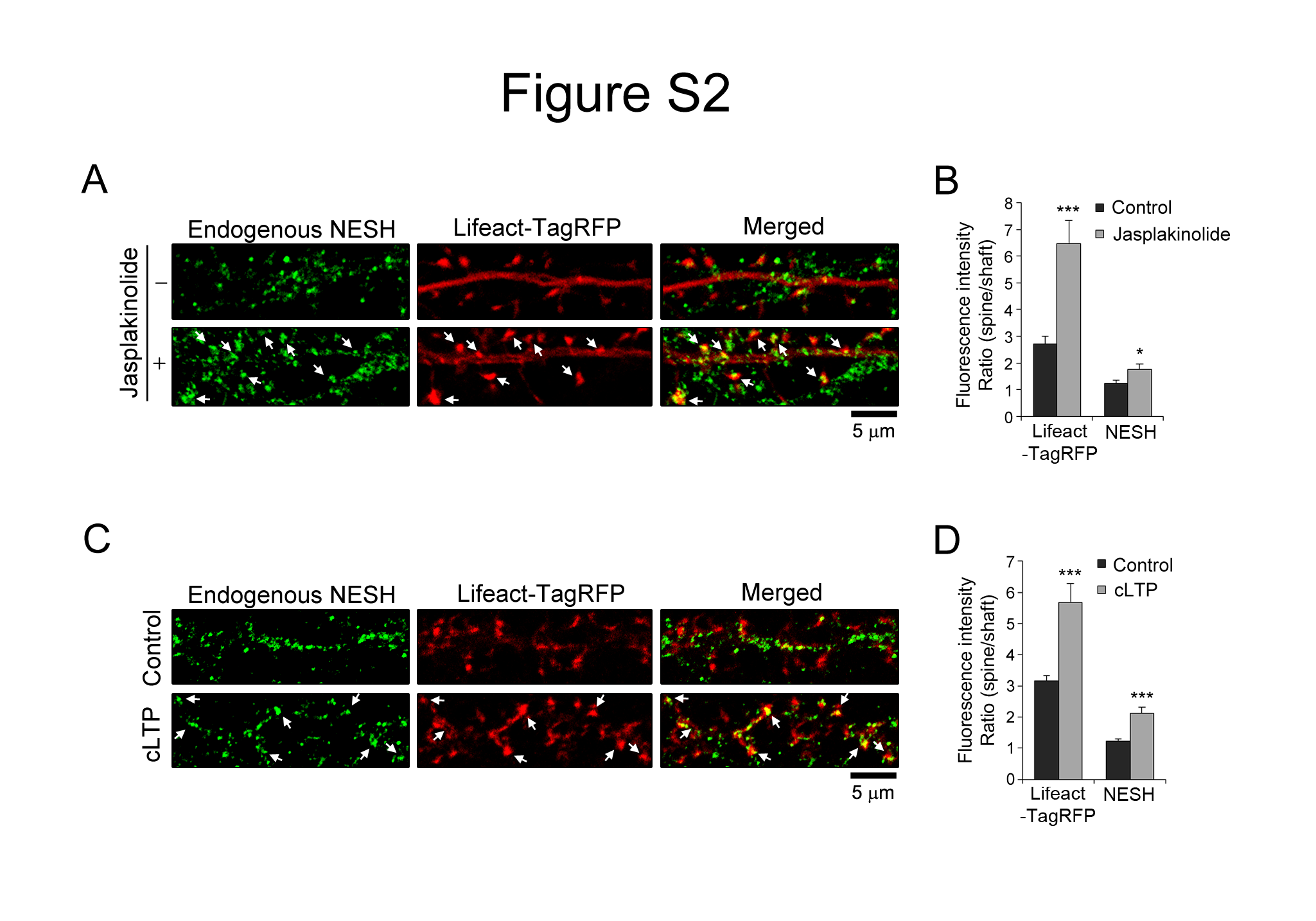

Supplement: Figure S2 — Synaptic translocation of endogenous NESH by F-actin stabilization and cLTP induction. (A) Hippocampal neurons were transfected with pLifeact-TagRFP at 10–12 DIV. pLifeact-TagRFP was used to visualize F-actin within cells. Transfected neurons at 16–18 DIV were treated with jasplakinolide (5 µM for 10 min), fixed, and stained with anti-NESH antibody. Colocalization between NESH and F-actin is indicated with white arrows in the merged image. (B) The intensity ratio (spine vs. shaft) was quantitatively analyzed from data obtained in Fig. S2A (N = 12 neurons for control, N = 19 neurons for jasplakinolide). (C) Synaptic translocation of endogenous NESH was examined during LTP. Hippocampal neurons at 10–12 DIV was transfected with pLifeact-TagRFP. After cLTP induction at 16–18 DIV, transfected neurons were fixed and stained with anti-NESH antibody, and NESH localization examined. White arrows in merged image indicate colocalization between NESH and F-actin. (D) Analysis of the fluorescence intensity ratio in dendritic spine vs. shaft from data obtained in Fig. S2C (N = 21 neurons for each condition). Data are presented as means ± SEM. *p<0.05, ***p<0.001. (TIF) [file pone.0034514.s002.tif]
